# A Narrative Review of Spinopelvic Alignment Changes After Total Hip Arthroplasty

**DOI:** 10.3390/jcm15062228

**Published:** 2026-03-15

**Authors:** Hiroyuki Ike, Hyonmin Choe, Naomi Kobayashi, Ken Kumagai, Yutaka Inaba

**Affiliations:** 1 Department of Orthopaedic Surgery, Yokohama City University, Yokohama 236-0004, Japan; hike@yokohama-cu.ac.jp (H.I.); hyonmin@hotmail.com (H.C.); 2 Department of Orthopaedic Surgery, Yokohama City University Medical Center, Yokohama 232-0024, Japan; naomik58@aol.com (N.K.); kumagai@yokohama-cu.ac.jp (K.K.)

**Keywords:** spinopelvic alignment, pelvic tilt, total hip arthroplasty (THA)

## Abstract

Total hip arthroplasty (THA) reliably restores function, yet instability remains a clinically relevant complication. Increasing evidence indicates that postoperative stability is strongly influenced by the dynamic spine–pelvis–hip interaction, which modulates functional acetabular orientation across postures. This narrative review summarizes current evidence on postoperative spinopelvic alignment changes after THA with emphasis on temporal patterns, underlying mechanisms, and predictive factors. Early after THA, restoration of hip motion can partially normalize hip-driven compensatory patterns, however substantial interindividual variability persists. Mid- to long-term follow-up shows that pelvic orientation continues to evolve, particularly progressive posterior pelvic tilt in standing, largely driven by aging and spinal degeneration, with acceleration in older patients and those with spinal pathology. Prediction of postoperative pelvic behavior requires integrated assessment of pelvic orientation, spinal alignment and mobility, contralateral hip status, and whether imbalance is hip-driven versus spine-driven. Although classification- and model-based approaches can estimate postoperative pelvic tilt, clinically meaningful prediction uncertainty remains, supporting a strategy focused on risk stratification and adaptive preoperative planning.

## 1. Introduction

Total hip arthroplasty (THA) is one of the most successful surgical procedures for restoring pain-free mobility and improving quality of life in patients with end-stage hip disease. However, dislocation remain clinically significant complications despite excellent long-term implant survivorship [1,2,3]. In particular, an increased risk of dislocation has been associated with abnormalities in the dynamic interaction between the spine, pelvis, and hip—often referred to as spinopelvic alignment—which plays an important role in determining functional acetabular orientation following THA [4,5,6,7].

**Figure 1:**
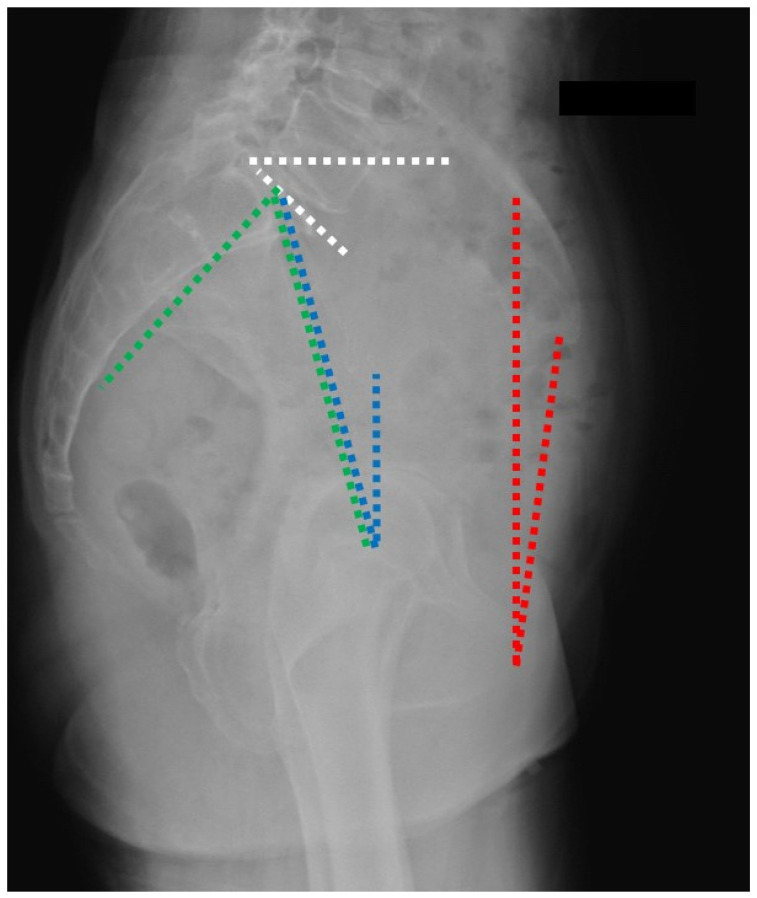
The spinopelvic alignment parameters: anterior pelvic plane tilt (red); sacral slope (white); pelvic tilt (blue); pelvic incidence (green).

Spinopelvic alignment is not static (Figure 1). Postural changes from standing to sitting produce coordinated motion of the spine and pelvis, altering acetabular orientation in functional positions [8,9,10,11]. Degenerative hip disease can further modify this relationship through compensatory mechanisms such as reduced hip extension, increased anterior pelvic tilt, and lumbar hyperlordosis, whereas surgical restoration of hip motion after THA may lead to both immediate and delayed changes in spinopelvic alignment [12,13]. These postoperative adaptations influence functional cup orientation, impingement risk, and the likelihood of instability, making spinopelvic considerations increasingly important in contemporary THA planning [14].

Previous studies have shown that THA can induce measurable changes in spinopelvic alignment, particularly in the early postoperative period [15,16,17]. Short-term improvements in hip range of motion (ROM) and pain relief may promote partial normalization of compensatory pelvic and spinal postures, but long-term adaptations appear more complex and heterogeneous, being influenced by spinal pathology, spinopelvic stiffness, and prior spine surgery [13]. Although numerous observational studies have investigated these phenomena, reported findings are inconsistent, partly because of variability in measurement methods, follow-up duration, and patient populations.

Most of the existing literature has focused on static, preoperative classifications of spinopelvic mobility or early postoperative change. In contrast, comparatively little attention has been paid to how spinopelvic alignment evolves over the long term after THA, and how these temporal changes differ between the short-term postoperative phase and long-term follow-up. Furthermore, the predictive factors underlying favorable normalization, persistent malalignment, or progressive deterioration of spinopelvic alignment remain poorly understood, particularly in the growing population of elderly patients with concomitant hip and spinal pathology.

Although several narrative reviews have addressed hip–spine syndrome and functional cup orientation, a focused synthesis of postoperative spinopelvic alignment changes across different time frames remains limited. Existing reviews tend to emphasize surgical strategy rather than postoperative biomechanical adaptation, and few integrate short-term and long-term perspectives within an integrated framework.

The purpose of this narrative review is to address these gaps by summarizing current evidence on spinopelvic alignment changes following THA, with particular emphasis on (1) short-term postoperative alterations, (2) long-term alignment changes, and (3) factors that may predict these changes. Compared with previous reviews, this manuscript places greater emphasis on the temporal evolution and predictive implications of postoperative alignment changes. By integrating temporal patterns with patient- and surgery-related predictors, this review aims to clarify the biomechanical consequences of THA on the spinopelvic system and to provide clinically relevant insights for surgical planning, risk stratification, and future research. Ultimately, a deeper understanding of postoperative spinopelvic behavior may enable more personalized THA strategies and improved long-term outcomes.

## 2. Materials and Methods

This narrative review synthesizes the current evidence concerning spinopelvic alignment and its postoperative evolution following THA. The objective was to provide an integrated and clinically meaningful overview of how spinopelvic parameters change after THA, how these changes can be predicted, and how they should inform contemporary surgical decision-making.

The literature was reviewed with particular focus on three interrelated domains:

(1) temporal patterns of spinopelvic alignment change after THA, including early, mid-term, and long-term postoperative phases;
(2) biomechanical mechanisms underlying spinopelvic adaptation, with emphasis on the distinction between hip-driven and spine-driven sagittal imbalance;
(3) predictive models and classification systems for postoperative pelvic tilt and mobility.

**Figure 2:**
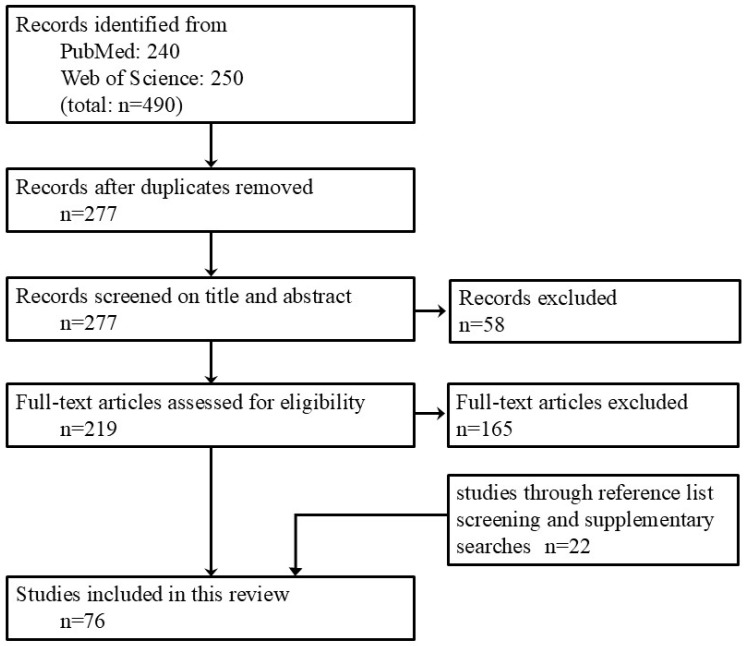
Flow diagram of study selection.

Studies were eligible if they (1) investigated changes in spinopelvic parameters following THA, (2) included quantitative radiographic and/or clinical measurements. Following title and abstract screening, 219 articles met the inclusion criteria and were selected for full-text review, of which 54 met the inclusion criteria and were included in the analysis. In addition, 22 relevant studies were identified through reference list screening and supplementary searches, resulting in a total of 76 articles included in the final analysis (Figure 2). The detailed search strategy is provided in the Supplementary Materials.

## 3. Immediate and Early Postoperative Spinopelvic Alignment Changes

### 3.1. Pathologic Alteration of Spinopelvic Alignment Induced by Hip Disease

Patients with end-stage hip disease frequently develop abnormal spinopelvic characteristics as compensatory responses to restricted hip motion, including anterior or posterior pelvic tilt, compensatory lumbar motion, and altered sagittal alignment. These adaptations are clinically important because they affect functional acetabular orientation and instability risk after THA.

In a prospective case–control study by Innmann et al., patients with hip osteoarthritis demonstrated significantly reduced hip flexion during sitting, accompanied by compensatory increases in posterior pelvic tilt and lumbar spine motion, consistent with spinopelvic hypermobility prior to THA [18]. One year after THA, preoperative differences in spinopelvic parameters disappeared. Hip flexion during sitting, magnitude of pelvic tilt change, and lumbar motion were comparable to those of age-, sex-, and BMI-matched healthy controls. Importantly, the prevalence of spinopelvic hypermobility was markedly reduced postoperatively, whereas static standing alignment parameters, such as sacral slope (SS), pelvic tilt (PT), and pelvic incidence–lumbar lordosis (PI–LL) mismatch, demonstrated minimal change.

Complementing these results, a retrospective cohort study focusing on lumbar hyperlordosis evaluated sagittal alignment, low back pain (LBP), and clinical outcomes following THA [19]. Lumbar hyperlordosis, defined by a PI–LL mismatch < −9°, was present in approximately 14% of patients undergoing THA. Preoperatively, these patients exhibited a unique sagittal alignment pattern with anterior pelvic tilt, exaggerated lumbar lordosis (LL), and a sigmoid-shaped spinal curvature, reflecting adaptive compensation for hip pathology. Following THA, pelvis tilted posteriorly and LL decreased, with significantly greater alignment correction observed in the hyperlordosis group, supporting the concept that lumbar hyperlordosis represents a flexible and adaptive condition rather than irreversible spinal disease. Despite substantial radiographic changes, there were no significant differences between the two groups regarding pre- and postoperative VAS for LBP and clinical outcomes.

Collectively, these studies indicate that some preoperative spinopelvic abnormalities observed in patients with hip osteoarthritis are hip-driven, functional adaptations rather than caused by fixed spinal deformities. In line with this concept, Thummala et al. demonstrated that hip-preserving and reconstructive procedures, including THA, periacetabular osteotomy, and hip arthroscopy, result in a significant reduction in standing pelvic tilt, while postoperative pelvic orientation remains strongly correlated with preoperative tilt [20]. Their findings further support the notion that abnormal pelvic alignment associated with hip disease is largely modifiable through restoration of hip mechanics rather than representing irreversible spinal pathology. Accordingly, careful distinction between a reversible spinopelvic alignment secondary to hip pathology and a rigid spinal alignment secondary to degenerative changes is essential.

### 3.2. Dynamic Changes in Spinopelvic Motion and Alignment Following THA

Numerous studies indicates that spinopelvic characteristics are not fixed after THA, but instead change in a posture-dependent and time-dependent manner [16,17,21,22,23]. A study evaluating spinopelvic motion in sitting, supine, and standing positions showed that clinically meaningful changes in SS (>±7°) occurred most frequently in sitting, affecting 44.3% of hips at one year after THA, compared with 21.6% in supine and only 7.9% in standing [24]. Furthermore, abnormal preoperative mobility patterns were not fixed: both stiff and hypermobile spinopelvic patterns partially normalized, with a distinct postoperative shift toward normal mobility.

In a large prospective cohort, Pour et al. demonstrated that although mean standing spinopelvic parameters remained relatively stable, approximately 25% of patients exhibited a change in standing SS of ≥7°, particularly in the early postoperative period [23]. Standing alignment proved more consistent over time, whereas relaxed-seated measurements showed substantial variability, limiting their reliability for long-term prediction of functional cup orientation.

Importantly, even in patients with normal preoperative sagittal alignment, THA itself altered spinopelvic mechanics. Qoreishy et al. reported a significant early postoperative reduction in SS, which was associated with short-term functional improvement [25]. Vergari et al. demonstrated that improvements in quality of life after THA are associated with pelvic tilt and pelvic range of motion (ΔSS_flexion-extension_) [26]. Their findings indicate that functional spinopelvic mobility, rather than static alignment alone, contributes meaningfully to postoperative patient satisfaction and daily function. Extending these observations to surgical strategy, Hagiwara et al. demonstrated that acetabular component orientation after THA changes dynamically according to spinopelvic classification [27]. Patients with normal spinal alignment and stiff pelvis had lower cup anteinclination (AI) in sitting.

At the whole-spine level, Haffer et al. demonstrated that THA induces measurable changes in sagittal spinopelvic alignment, primarily through reduction in posterior pelvic tilt, while coronal alignment remains unaffected [28]. Patients with preexisting sagittal spinal imbalance showed persistent compensatory patterns despite partial improvement, identifying them as a high-risk group for anterior dislocation in standing. Consistent with this risk phenotype, Konishi et al. showed that after THA, patients with flatback deformity maintain persistently posterior pelvic tilt [29]. Moreover, patients with stiff spinopelvic mobility compensate by increasing hip flexion–extension during chair-rising, a mechanism that may increase impingement risk and suggests the need for individualized preoperative planning based on spinopelvic alignment and mobility.

These studies indicate that spinopelvic alignment after THA is dynamic, posture-dependent, and partially reversible. Distinguishing flexible, hip-driven adaptations from fixed imbalance due to spinal deformity or fixation is therefore essential for accurate risk stratification, preoperative planning, and optimization of THA outcomes.

### 3.3. Spinopelvic Hypermobility as a Dynamic and Hip-Driven Condition

Recent evidence suggests that preoperative spinopelvic hypermobility should not be regarded as a fixed spinal pathology, as it frequently improves after treatment of the hip joint.

In a retrospective study by Sculco et al., 136 patients with preoperative spinopelvic hypermobility—defined as a standing-to-sittingSS change (ΔSS_stand-relaxed seated_) of ≥30°—were followed after primary THA [30]. At one year postoperatively, 95% of patients demonstrated normalization of spinopelvic mobility, with mean ΔSS_stand-relaxed seated_ decreasing from 36.4° preoperatively to 21.4°. Persistent hypermobility was observed in only 5% of patients, and the only significant predictor was advanced osteoarthritis of the contralateral hip, indicating that unresolved hip pathology plays a key role in maintaining abnormal spinopelvic motion.

Extending these findings, Windsor et al. evaluated patients with preoperative hypermobility undergoing staged bilateral THA [31]. Hypermobility resolved in only 29% after the first THA but normalized in 98% of patients one year after the second THA, with a marked reduction in ΔSS_stand-sit_ and a substantial decrease in functional acetabular anteversion in sitting. The authors cautioned that reducing acetabular anteversion solely based on preoperative hypermobility may be unfavorable, as postoperative normalization could result in insufficient functional anteversion and increased instability risk.

These studies indicate that preoperative spinopelvic hypermobility is often hip-driven and reversible, emphasizing the need for caution when modifying component positioning based solely on preoperative spinopelvic hypermobility.

### 3.4. Contributing Factors to Postoperative Pelvic Tilt

Ishida et al. conducted a prospective clinical study to clarify the chronological behavior of pelvic tilt (PT) after THA and to determine how preoperative pelvic orientation is associated with postoperative PT changes [15]. Using the anterior pelvic plane tilt (APPt) measured on lateral radiographs, the authors demonstrated a strong relationship between preoperative pelvic alignment and postoperative APPt change. Patients with severe anterior pelvic tilt preoperatively showed the largest posterior correction after THA, whereas those with posterior tilt did not show significant changes. Importantly, most PT changes occurred within the first 3 months, followed by a more gradual progression thereafter, indicating an early adaptive response to restoration of hip motion.

Although Ishida et al. demonstrated that the direction of postoperative PT change is generally predictable, they also observed marked variability. Notably, some patients exhibited large-magnitude changes. These findings demonstrate the importance of identifying additional factors beyond preoperative pelvic orientation alone.

In this regard, Oetojo W et al. demonstrated that contralateral hip status significantly influences early postoperative PT change [32]. Patients with a normal contralateral hip experienced greater posterior pelvic tilt after THA, whereas those with advanced contralateral osteoarthritis or a previously replaced hip showed smaller changes. This finding suggests that PT change after THA reflects bilateral hip biomechanics.

Further advancing predictability, Tang et al. developed a patient-specific algorithm that accurately predicted one-year postoperative PT change based on sagittal alignment type, incorporating global balance and lumbar compensation patterns [33]. Their work demonstrates that postoperative PT behavior can be estimated quantitatively rather than inferred qualitatively. These studies indicate that postoperative pelvic tilt change is not random, but instead follows predictable patterns determined by preoperative pelvic orientation, contralateral hip condition, and sagittal alignment type.

Palit A et al. investigated the impact of PT on postoperative ROM and impingement mechanisms after THA [34]. Using 3D-CT–based models from 56 patients, the authors simulated hip motion under varying PTmeasured preoperatively and at 6 and 12 months postoperatively. PT demonstrated a strong correlation with flexion, extension, and internal rotation at 90° of flexion, while its effect on other movements was minimal. Each 10° increase in anterior PTresulted in approximately 10° reduction in flexion and internal rotation at 90° of flexion. Although postoperative PT changes were generally modest and stabilized after 6 months, individual variations led to significant functional ROM loss in a subset of patients. The study highlights the importance of incorporating patient-specific spinopelvic alignment into THA planning and evaluation.

### 3.5. Classifying Hip-Driven and Spine-Driven Imbalance

Buckland et al. demonstrated that preoperative classification of sagittal spinopelvic deformity is a decisive determinant of alignment change after THA [35]. By explicitly distinguishing hip-driven sagittal imbalance (“apparent deformity”) from spine-driven true thoracolumbar deformity, their study clarified why postoperative spinopelvic behavior varies substantially among patients traditionally grouped together as “high risk.” Patients with apparent deformity—defined by increased sagittal vertical axis (SVA) despite preserved PI–LL and T1 pelvic angle (TPA)—showed the largest postoperative improvements in both standing and sitting alignment. Patients presenting with an apparent deformity due to limited hip ROM exhibit the most substantial changes in both standing and sitting compared with those with thoracolumbar deformity or normal spinal alignment. In contrast, patients with thoracolumbar deformity showed the least change in spinopelvic parameters, likely due to the preexisting posterior pelvic tilt and spinal stiffness.

This classification-based framework is supported by Jain et al., who demonstrated that THA can improve global sagittal alignment, particularly by reducing SVA [36]. Importantly, the degree of improvement was greater in patients with high TPA before surgery. These results suggest global spinal alignment correction is most evident in patients whose imbalance can still respond to restoration of hip function.

Importantly, Zhang et al. provide a critical temporal exception to early postoperative models [37]. In patients with bilateral Crowe type IV developmental dysplasia of the hip, pelvic sagittal tilt does not change immediately after surgery but demonstrates a delayed posterior shift. Radiographic parameters showed no significant alteration in PTat 3 months postoperatively. In contrast, statistically significant posterior pelvic tilt emerged at 6 months and progressed further by 12 months, indicating that pelvic realignment is not an immediate consequence of the surgical procedure in this population. These findings suggest that postoperative pelvic tilt change reflects gradual soft-tissue remodeling and neuromuscular rebalancing.

Taken together, these studies indicate that many spinopelvic abnormalities observed in patients with hip osteoarthritis are hip-driven, functional adaptations rather than fixed spinal deformities. Following THA, the pelvis shows a posterior tilt by one year, and importantly, the majority of this pelvic tilt change occurs within the first 3 postoperative months. While individual variability exists, later changes from 3 to 12 months are generally modest, except in specific populations with severe deformity. Pelvic tilt change is not random; its direction and magnitude are influenced by preoperative pelvic orientation, contralateral hip condition, and sagittal alignment type. Because pelvic tilt substantially affects functional ROM and impingement risk, early postoperative pelvic realignment should be anticipated in THA planning.

### 3.6. Postural and Individual Variability of Pelvic Tilt After THA

Pelvic tilt after THA must be understood as a posture-dependent and patient-specific parameter rather than a static quantity. When examined at the level of group averages, multiple studies consistently report that pelvic tilt changes by only 2° to 3° in supine after THA [16,38,39]. Kamihata et al. investigated whether hip flexion contracture alters pelvic tilt in the supine position after THA. Release of contracture significantly reduced femoral flexion but resulted in minimal PT changes in supine [40]. In line with these observations, Pluchon et al. reported that mean PT in standing, sitting, and supine showed no significant change at 6 months after THA; however, they also demonstrated substantial intra- and inter-individual variability across day-to-day postures [41]. However, this population-level stability masks clinically meaningful differences between postures (supine, standing, sitting) and substantial intra- and inter-individual variability, which cannot be ignored in functional cup positioning.

Nishihara S. et al. demonstrated that pelvic orientation differs markedly across postures even before surgery [38]. Using CT-based 3D/2D matching, the authors showed that mean pelvic flexion angle was approximately 5° in supine, 3° in standing, and −29° in sitting, indicating pronounced posterior pelvic tilt in the sitting position. Importantly, despite wide interindividual variability, pelvic orientation within a given posture was highly reproducible within each patient and largely preserved one year after THA.

This concept of modest change at the cohort level was reinforced by Murphy et al. [39]. They showed that mean PT changed by less than 2° postoperatively in supine and did not change significantly in standing. More than 90% of patients exhibited postoperative PT changes of less than 5°, and preoperative PT strongly predicted postoperative PT. Another study by Kleeman-Forsthuber et al. showed that pelvic incidence (PI) alone does not predict spinal or pelvic mobility in THA patients [42]. Instability risk relates more to sagittal spinal deformity and mobility patterns than to PI in isolation. These findings indicate that postoperative pelvic tilt is closely associated with preoperative pelvic orientation.

However, Murphy et al. also emphasized that baseline pelvic tilt varied widely between individuals, questioning the appropriateness of the uniform acetabular target angles [39]. This observation is consistent with results of Maratt et al., which reported a mean standing APPt near neutral both before and after THA, yet found that 17% of patients exhibited ≥10° of anterior or posterior tilt preoperatively [43]. Biomechanical modeling in that study demonstrated that each degree of posterior pelvic tilt increased functional anteversion of acetabular component by approximately 0.74°.

Extending these findings to surgical decision-making, Dennis et al. demonstrated that individualized acetabular cup positioning based on preoperative spinopelvic mobility analysis significantly reduced prosthetic impingement compared with conventional safe-zone placement [44]. Importantly, bone-to-bone impingement remained frequent and largely independent of cup orientation, underscoring that variability in spinopelvic behavior—rather than component position alone—plays a central role in postoperative impingement risk.

The importance of measurement modality and posture was further clarified by an EOS-based study [45]. At the population level, mean standing PI, SS, and pelvic version did not change significantly after THA. Nevertheless, 35% of patients showed >5° change in SS, and 22.5% demonstrated clinically relevant changes in pelvic version. These findings show that pelvic orientation can change in a considerable number of patients, even when the average remains stable.

Evidence from EOS-based sagittal orientation studies in Japanese patients (196 cases) further complicates the notion of stability [46]. This study demonstrated an average posterior pelvic tilt of approximately 3° in standing within 3 months after THA, indicating a postoperative change in pelvic orientation. More importantly, the authors demonstrated substantial interindividual variability and showed that incorporating preoperative factors—such as age, LL, and pelvic tilt in both supine and standing positions—significantly improves the prediction of postoperative standing pelvic tilt. Morphological parameters such as PI alone were insufficient for prediction, indicating that anatomy-only models are limited.

Finally, Du et al. provide a critical temporal perspective in complex hips [47]. In Crowe III/IV developmental dysplasia, pelvic tilt followed a non-linear time course: no marked change was observed in the early postoperative period, followed by progressive posterior tilt by one year. Although this represents a specific patient population, it clearly demonstrates that time-dependent pelvic adaptation can be clinically significant, even when early postoperative measurements appear stable.

At the level of group averages, pelvic tilt after THA remains relatively stable, particularly in standing or supine positions [38,39,43]. However, posture-specific differences, individual variability, and longitudinal changes are clinically non-negligible, especially when considering functional acetabular orientation [46,47]. Therefore, pelvic tilt should not be treated as a fixed value but rather as a dynamic, posture-dependent parameter, requiring patient-specific assessment to optimize THA strategy.

### 3.7. Predictable Change and Risk Stratification of Spinopelvic Behavior After THA

Age is one of the influential factors for classifying spinopelvic behavior in patients undergoing THA. In a prospective EOS-based study of 197 primary THA patients, Haffer et al. showed that spinopelvic function follows clear age-dependent patterns: lumbar flexibility and pelvic mobility decline progressively with aging, while pelvic posterior tilt in standing and global sagittal imbalance increase [48]. Importantly, these age-related differences persisted after THA, and the authors provided age-adjusted reference values, supporting the concept that spinopelvic behavior is not random but systematically stratifiable, with age acting as a dominant factor that constrains postoperative adaptability. Consistent with this concept, Shirono et al. reported age-dependent changes in pelvic tilt in a large cohort of Japanese women, demonstrating progressive posterior pelvic tilt with advancing age while PI remained unchanged [49]. Their findings indicate that spinopelvic alignment evolve in predictable, age-related patterns.

Building on this framework, spinal alignment represents the next key skeletal determinant that shapes how each component of the spinopelvic complex behaves. Using the same prospective EOS cohort, Haffer et al. demonstrated that sagittal malalignment—defined by elevated C7-SVA, PI–LL mismatch, or specific Roussouly types—produces predictable, structured effects on component motions: sagittally imbalanced patients showed reduced lumbar flexibility (ΔLL), altered pelvic behavior (ΔPT), and compensatory changes in hip motion (ΔPFA), both before and shortly after THA [50]. In contrast, coronal balance had little effect on this sagittal spinopelvic alignment. Together, these two studies support a practical hierarchy for classification: age defines the baseline functional capacity, which is then refined by sagittal spinal alignment that redistributes motion demands across the lumbar spine, pelvis, and hip.

Recent studies have clarified that accurate prediction of postoperative spinopelvic behavior after THA depends on detailed preoperative assessment of spinal and pelvic mobility, rather than on single static measurements. Watanabe et al. demonstrated that postoperative pelvic stiffness cannot be reliably inferred from preoperative pelvic mobility alone [51]. Although pelvic mobility defined by ΔSS_stand-relaxed seated_ decreased on average after THA, a substantial proportion of patients developed postoperative stiff pelvis despite having normal or hypermobile pelvis preoperatively. Crucially, reduced preoperative lumbar mobility (ΔLL) and lower standing LL emerged as key predictors, especially among patients who appeared mobile before surgery. These findings established lumbar alignment and lumbar flexibility as critical determinants of postoperative pelvic behavior.

Homma et al. reinforced this concept by showing that pelvic mobility before and after THA is not equivalent and exhibits wide interindividual variability [52]. Using ΔSS_stand-relaxed seated_ as a continuous parameter, they demonstrated a right-skewed distribution both pre- and postoperatively, with pelvic mobility generally decreasing after THA. Importantly, postoperative pelvic mobility was influenced by multiple preoperative factors, including standing and sitting SS, lumbar scoliosis, and contralateral hip pathology. This study emphasized that postoperative pelvic motion is multifactorial and cannot be predicted by ΔSS_stand-relaxed seated_ alone, demonstrating the necessity of detailed preoperative assessment.

Kim et al. expanded the framework by elucidating the kinematic chain linking hip, pelvic, and lumbar motion [53]. Their prospective analysis across multiple postural conditions showed that restoration of hip mobility after THA leads to a redistribution of sagittal motion: hip motion increases, while pelvic and lumbar motion decrease. Strong correlations between changes in hip motion (ΔPFA), pelvic mobility (ΔSS _stand-flexed seated_ and ΔSS_stand-relaxed seated_), and lumbar mobility (ΔLL) confirmed that spinopelvic behavior is dynamically interdependent. Importantly, the magnitude and pattern of these interactions varied depending on postural conditions, highlighting that mobility assessment is posture-specific.

The study by Berliner et al. focused on postoperative sitting pelvic position, a key determinant of functional acetabular anteversion [54]. They demonstrated marked interindividual variability one year after THA and identified preoperative ΔSS_stand-relaxed seated_ and the presence or absence of lumbar degenerative disc disease as major predictors. Patients with degenerative spines exhibited fixed, predictable pelvic behavior, whereas those with normal spines showed greater postoperative variability due to release of hip-driven compensatory mechanisms.

A prospective matched-pair study by Muellner et al. examined whether a pre-existing contralateral THA affects spinopelvic mobility in patients undergoing primary THA [55]. Forty-four patients with a prior contralateral THA were matched to 44 controls without prior THA and evaluated using standing and sitting EOS imaging before and shortly after surgery. Preoperatively, patients with an existing THA showed greater pelvic mobility and lumbar flexibility, whereas controls demonstrated restricted pelvic motion with compensatory increased hip motion. After THA, pelvic and lumbar mobility increased and hip motion decreased in both groups, indicating redistribution of motion. The authors conclude that a pre-existing THA significantly influences spinopelvic kinematics and may be associated with more favorable mobility patterns.

The heterogeneity in study design, imaging modality, and patient population limits direct comparability and requires cautious interpretation (Table 1). Nevertheless, the available evidence suggests that anticipating postoperative pelvic stiffness and alignment changes requires preoperative evaluation of mobility across multiple segments and postures. Pelvic behavior after THA is determined not only by baseline mobility but also by lumbar alignment, hip motion, spinal degeneration, and the posture in which mobility is assessed. By preoperatively screening patients for abnormal spine–hip relationships, it is possible to refine THA surgical planning, which may improve outcomes [56]. Conventional acetabular target zones, including the Lewinnek safe zone, may not be applicable to patients with abnormal spinopelvic mobility [57].

## 4. Mid- to Long-Term Spinopelvic Alignment Changes

When short-term postoperative adaptation is excluded, the available literature demonstrates that pelvic tilt after THA continues to evolve over mid- to long-term follow-up, with the magnitude and pattern of progression strongly dependent on observation duration and patient posture. Studies extending beyond 2 years consistently show that pelvic orientation is not a static parameter after THA, but rather reflects an ongoing interaction between aging, spinal degeneration, and posture-dependent biomechanics.

### 4.1. Mid-Term Spinopelvic Alignment Changes

Mid-term studies with follow-up periods of 2 to 5 years first revealed that pelvic tilt does not simply stabilize once early postoperative changes have resolved. Taki et al. examined pelvic tilt in standing and supine positions up to 4 years after THA [17]. Although mean PT values appeared relatively stable after the early postoperative phase, the authors demonstrated a progressive increase in the difference between standing and supine pelvic tilt (dPA) over time. Importantly, the proportion of patients with a large postural discrepancy (dPA > 10°) increased steadily during follow-up.

A prospective study by Blondel B et al. demonstrated that standing pelvic tilt remained stable after THA within a selected patient population and over a mid-term follow up of 3 years [63]. In 50 patients with unilateral hip osteoarthritis, without bilateral disease or severe spinal degeneration, standing pelvic tilt showed no significant change between preoperative assessment and postoperative follow up, with most patients exhibiting less than 5° of variation.

Extending observation to 5 years, Suzuki et al. provided further evidence that pelvic tilt progression persists beyond the mid-term window [16]. The authors quantified the maximum posterior pelvic tilt change between the preoperative supine position and the standing position at 5 years postoperatively. While most patients demonstrated moderate posterior tilt progression, approximately 8% exhibited marked and progressive posterior pelvic tilt exceeding 20°, which continued to worsen throughout the five-year follow-up. These patients were characterized by older age and reduced preoperative LL, indicating limited spinal compensatory capacity. Notably, hip clinical outcomes improved uniformly across all groups, emphasizing that progressive pelvic tilt can occur independently of hip-specific functional recovery.

### 4.2. Long-Term Spinopelvic Alignment Changes

Longer follow-up studies have consistently shown that pelvic tilt progression becomes more pronounced and posture-specific over time. Tamura et al. investigated pelvic sagittal inclination over a 10-year period after THA and identified a clear divergence between supine and standing measurements [21]. Supine pelvic orientation remained largely unchanged beyond the early postoperative period, whereas standing pelvic tilt demonstrated a gradual but substantial posterior shift over time. As a result, nearly half of the cohort developed a standing posterior tilt exceeding 10° relative to the supine position at 10 years. This posture-specific progression strongly suggests that long-term pelvic tilt changes are driven primarily by age-related spinal changes affecting upright alignment, rather than by continued remodeling of hip mechanics.

The temporal characteristics of this progression were further clarified by Katsura et al., who examined standing pelvic tilt over more than 10 years and demonstrated that posterior pelvic tilt does not progress at a constant rate [64]. After the mid-term period, pelvic tilt advanced slowly for several years but accelerated again in older patients, particularly after approximately 75 years of age. This late-phase acceleration was most pronounced in patients with fragility fractures.

García-Rey et al. reported that female sex, age over 65 years were associated with sacro-femoral-pubic angle [65]. Female and older patients are more prone to developing sagittal deformity due to aging and possible decreases in bone mineral density. In addition, Okanoue et al. showed that preoperative posterior pelvic tilt in the standing position and vertebral fractures during follow-up as significant factors of increased functional acetabular anteversion [66].

Kromka et al. evaluated changes in pelvic tilt and functional acetabular position in patients undergoing THA or surface replacement arthroplasty, with a minimum 10-year follow-up [67]. Over 10 years, mean pelvic tilt shifted posteriorly by −9.87°, corresponding to an estimated increase of 1.97° in inclination and 7.90° in anteversion. Younger patients (45–50 years) demonstrated significantly greater posterior tilt progression than those aged 55–60 years (−11.50° vs. −8.25°), suggesting age-related differences in pelvic tilt over the long term.

Very long-term data extending to two decades further reinforce this concept. In a 20-year longitudinal study, pelvic tilt was evaluated in supine, standing, and sitting positions following THA [22]. Over time, both supine and standing pelvic tilt shifted posteriorly, but the magnitude of change was consistently greater in the standing position, while sitting pelvic tilt remained largely unchanged. Moreover, the standing-to-sitting pelvic shift decreased with advancing age, indicating increasing pelvic stiffness. Preoperative posture-dependent pelvic behavior was a strong predictor of long-term standing alignment, underscoring the importance of baseline spinopelvic characteristics in determining decades-long pelvic orientation.

Similarly, Chen et al. reported sustained posterior pelvic reorientation at a minimum of 18 years after THA [68]. More than 20% of patients demonstrated pelvic tilt changes exceeding 10°, and these changes were associated with corresponding alterations in functional acetabular orientation. Female sex and older age were linked to greater pelvic tilt at final follow-up.

### 4.3. Special Populations with Altered Spinopelvic Mechanics

In patients with severe bilateral Crowe type IV developmental dysplasia, spinopelvic malalignment represents a hip driven compensatory phenomenon rather than a primary spinal disorder. In a cohort of relatively young patients undergoing simultaneous bilateral THA, Can et al. demonstrated that restoration of the hip center resulted in marked improvement in pelvic orientation, with pelvic tilt shifting toward neutral values and relief of low back pain [69]. Importantly, LL showed little postoperative change, suggesting that symptom improvement was primarily related to correction of pelvic orientation and hip centers rather than normalization of lumbar sagittal alignment.

Further evidence supporting the reversibility of spinopelvic compensation was provided by Morimoto et al. in patients with bilateral highly dislocated hips treated with subtrochanteric shortening THA [70]. In this older cohort, excessive pelvic anterior tilt and lumbar hyperlordosis were significantly reduced after anatomical reconstruction of the hip center, with normalization of segmental lumbar curvature when spinal flexibility was preserved. These findings indicate that abnormal spinopelvic alignment in severe hip dislocation reflects adaptive mechanisms secondary to loss of the hip center.

In contrast to age-related or degenerative changes in pelvic tilt over time, spinal fixation or inflammatory ankylosis primarily alters spinopelvic mobility itself rather than inducing progressive pelvic tilt change.

In a case–control study reported by Grammatopoulos et al., patients who underwent THA in the setting of prior spinal arthrodesis demonstrated markedly altered spinopelvic mechanics compared with matched controls [71]. Although standing pelvic tilt tended to be more posterior, the defining abnormality was excessive dynamic pelvic motion, with spinopelvic hypermobility being five times more prevalent. Importantly, hypermobility—not stiffness—was the only biomechanical factor independently associated with inferior patient-reported outcomes and recurrent instability, even when acetabular components were positioned within traditional safe zones.

A different but equally distinct pattern was observed in patients with ankylosing spondylitis, as reported by Oommen et al. [72]. In this population, spinopelvic mechanics were dominated by severe stiffness, most commonly a persistent “stuck sitting” pattern, which remained unchanged after THA. While hip motion and clinical outcomes improved substantially, pelvic and spinal motion did not recover, indicating that THA does not normalize spinopelvic mobility in ankylosed spines.

Taken together, mid- to long-term studies consistently demonstrate that pelvic tilt after THA continues to progress over years and decades, particularly in the standing position. Mid-term follow-up identifies increasing posture-dependent divergence, while long-term observations reveal progressive posterior tilt driven predominantly by aging and spinal degeneration; this progression has also been associated with female sex, vertebral fractures during follow-up, and preoperative posterior pelvic tilt in the standing position (Table 2). The progression does not follow a linear pattern and may accelerate with advancing age or in the presence of spinal pathology. Pelvic tilt should be conceptualized not as a fixed postoperative parameter, but as a time-dependent biomechanical process that evolves across the lifespan. Given that posterior pelvic tilt tends to progress with aging, excessive cup inclination or anteversion at the time of surgery may predispose to unfavorable long-term outcomes; therefore, careful consideration is required during component positioning.

## 5. Prediction of Postoperative Spinopelvic Alignment and Clinical Implications

Postoperative changes in pelvic tilt after THA have emerged as a critical determinant of functional acetabular component position and late instability. Rather than being static, pelvic orientation evolve over time, and recent studies have focused on whether these changes can be predicted preoperatively, how reliable such predictions are, and which biomechanical mechanisms underlie postoperative variability.

Early evidence supporting the predictability and clinical relevance of PT after THA was provided by Murphy et al. [39]. Using CT based image matching and standing/supine radiographs, the authors demonstrated that PT changes minimally after THA in most patients. More importantly, preoperative PT strongly predicted postoperative PT in both standing and supine positions. These findings established a key conceptual framework: pelvic orientation is largely patient specific and remains stable. Therefore, preoperative assessment of PT should be regarded as the starting point for predicting postoperative pelvic orientation. This work provides an essential foundation for subsequent studies on PT prediction.

The clinical importance of predicting pelvic orientation was further clarified by a later study that quantified the relationship between PT and functional acetabular component orientation [43]. Together, these studies suggest that PT is partly predictable and clinically important in personalized THA planning.

Ishida et al. demonstrated that postoperative changes in pelvic tilt are influenced by preoperative orientation [15]. Specifically, hips with marked preoperative anterior pelvic tilt tended to undergo a posterior shift after THA, whereas the magnitude and direction of change varied according to patient age and baseline postural characteristics. Pelvic tilt behavior cannot be adequately described by a single, universal predictive rule. Their study reinforces that pelvic tilt prediction is feasible but inherently constrained by interindividual variability, thereby justifying further investigation into the mechanisms underlying prediction failure and the identification of high-risk or outlier subgroups.

Homma et al. showed in a retrospective cohort that postoperative ΔSS_stand-relaxed seated_ is governed by multiple preoperative factors, including standing and sitting SS, LL, and contralateral hip pathology, in addition to preoperative ΔSS_stand-relaxed seated_ [52]. This study demonstrates wide interindividual variability and confirms that pelvic mobility often decreases after THA.

Windsor et al. further reinforced this concept in patients undergoing staged bilateral THA [31]. They showed a stepwise and nearly universal resolution of hypermobility after treatment of both hips, with normalization in 98% of patients one year after the second THA. The reduction in ΔSS_stand-sit_ and functional anteversion supports the notion that spinopelvic hypermobility is predominantly hip-driven.

Buckland et al. provided a conceptual framework showing that postoperative pelvic change after THA is largely determined by whether sagittal imbalance is hip driven or spine driven [35]. Using standing and sitting EOS radiographs, they demonstrated that patients with apparent deformity, in whom sagittal imbalance is caused primarily by hip flexion contracture, exhibit substantial postoperative changes in spinopelvic alignment, including normalization of pelvic mobility and reduction in sagittal vertical axis. In contrast, patients with true thoracolumbar deformity showed minimal change in standing and sitting PT, reflecting fixed spinal stiffness and limited compensatory capacity. Based on the same rationale, Tang et al. translated sagittal alignment concepts into a quantitative, patient-specific prediction model [33]. They aimed to establish a reliable method to predict individual postoperative changes rather than relying on population averages. By classifying patients according to global alignment patterns and applying either an SVA-driven or LL–driven algorithm, they predicted one-year postoperative standing PT with a mean absolute error of approximately 3°. Their results confirm that, once the dominant compensatory mechanism is identified, postoperative PT can be predicted with clinically acceptable accuracy.

In a large retrospective study, Fujii et al. addressed the long-term behavior of pelvic orientation by developing a machine-learning model to predict changes in pelvic flexion angle 5 years after THA [61]. Analyzing 415 hips in 343 patients with comprehensive preoperative imaging, the authors demonstrated that long-term posterior pelvic tilt is not rare: approximately 11 percent of hips showed marked posterior tilt greater than 20°, with some exceeding 40° beween preoperative supine and standing at 5 years. Importantly, predictive performance using preoperative variables alone was moderate, but accuracy improved substantially when early postoperative data were incorporated. In particular, the change in pelvic flexion angle at 1 year emerged as the strongest predictor of 5-year pelvic behavior. Early postoperative alignment contains critical information about long-term evolution. At the same time, the study highlights that meaningful pelvic change occurs in a non-negligible subset of patients.

While Fujii et al. demonstrated that prediction is feasible, Fischer et al. examined the practical implications of prediction uncertainty for patient-specific THA planning [58]. Using detailed three-dimensional reconstructions in 196 patients, they quantified uncertainty in predicting postoperative standing PT. Even with multivariable regression models incorporating age, preoperative PT, and LL, the 95 percent prediction interval for postoperative PT averaged approximately 14°. When this uncertainty was propagated into ROM-based and load-based acetabular target zones, the clinically usable safe zones were dramatically reduced. In many patients, the combined ROM and load-based target zone nearly disappeared once realistic prediction error was considered. This work underscores that even statistically sound prediction models may have limited clinical robustness when applied to individualized cup positioning. Together with Fujii et al., it suggests that long-term pelvic change is not only patient-specific but also inherently uncertain, placing constraints on highly individualized preoperative planning strategies.

An explanation for this variability is provided by the prospective radiographic study with evaluation of standing and sitting alignment [54]. This study demonstrated that restoration of hip motion redistributes movement within the lumbar–pelvic–femoral complex, but the direction and magnitude of pelvic change depend strongly on spinal condition. Patients with multilevel lumbar degenerative disk disease exhibited relatively fixed pelvic alignment before and after THA, resulting in more predictable postoperative pelvic position. In contrast, patients with relatively normal and flexible spines showed wide variability in postoperative sitting pelvic tilt, with both anterior and posterior shifts observed. Preoperative ΔSS_stand-relaxed seated_ and the absence of spinal degeneration were key predictors of postoperative pelvic behavior. These findings clarify why prediction models perform variably across populations: in patients with flexible spines, pelvic orientation is influenced by complex compensatory mechanisms that may readjust after hip reconstruction, whereas degenerative spines constrain pelvic motion and limit postoperative change.

Heckmann et al. demonstrated that postoperative pelvic tilt cannot be assumed to mirror preoperative supine alignment [59]. In a cohort of 933 patients, approximately 10% exhibited excessive posterior pelvic tilt when transitioning from preoperative supine to postoperative standing, exceeding a threshold associated with a clinically meaningful increase in functional acetabular anteversion. Importantly, this high-risk subgroup was predictable preoperatively, as excessive posterior tilt was strongly associated with a large preoperative supine-to-standing PT difference. These findings underscore that failure to anticipate this pattern may lead to unrecognized functional over-anteversion and anterior instability, particularly in supine-based surgical approaches.

This chapter shows that pelvic tilt after THA is neither random nor completely fixed. In many patients, pelvic tilt remains close to its preoperative state, which makes preoperative assessment an essential starting point for prediction. However, multiple studies demonstrate that meaningful deviations do occur and follow recognizable patterns. The direction and magnitude of change depend on factors such as preoperative pelvic orientation, spinal mobility, hip contracture, contralateral hip condition, and whether sagittal imbalance is driven mainly by the hip or the spine. Recommendations for cup positioning are therefore switching from a systematic to a patient-specific approach [60].

Spinopelvic hypermobility is now understood as reversible, hip-driven adaptations that often normalize after adequate treatment of hip pathology, especially after bilateral THA. Studies on prediction uncertainty make it clear that exact prediction is not always possible and that small errors may have large clinical effects [58,59]. Moreover, spinopelvic risk factors and mobility are associated with patient reported outcomes [60,62]. Future work should therefore focus less on exact prediction and more on identifying high-risk patients, integrating whole-spine and bilateral hip assessment, and adjusting surgical planning and follow-up to avoid excessive posterior pelvic tilt changes after THA.

**Figure 3:**
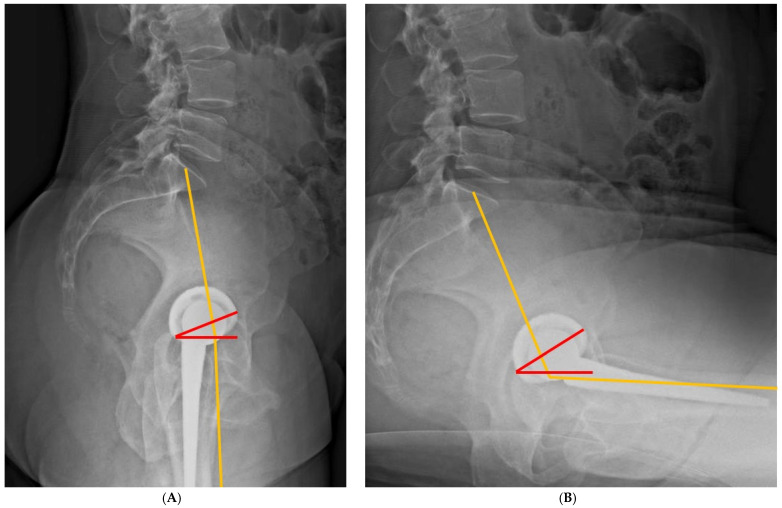
(**A**) Combined sagittal index (CSI) in the standing position; (**B**) CSI in the sitting position. CSI is defined as the sum of acetabular anteinclination (AI, red line) and pelvic femoral angle (PFA, orange line) (CSI = AI + PFA). Standing outliers were defined as patients with a standing CSI > 243° [1,75].

Based on these findings, several practical implications for surgical planning can be identified. First, preoperative evaluation should incorporate standing pelvic orientation and, when indicated, dynamic spinopelvic assessment in the relax-seated or flexed-seated position [4,5,6,53]. Second, patients should be stratified according to spinopelvic characteristics, including spinopelvic mobility patterns, sagittal spinal alignment, PI–LL mismatch, and contralateral hip pathology [13,56,57,73]. Third, acetabular component positioning should be planned with reference to functional alignment parameters, rather than adherence to historical static safe-zone concepts [1,74,75]. The combined sagittal index (CSI) is defined as the sum of the pelvic femoral angle (PFA) and acetabular AI (Figure 3). A decreased sitting CSI was associated with posterior dislocation, whereas an increased standing CSI was associated with anterior dislocation.

The presence of multiple spinopelvic risk factors, particularly a standing APPt ≤ −10°, is associated with significantly lower postoperative functional outcomes [60]. In such patients, spinopelvic evaluation and patient-specific surgical planning are strongly recommended. Robotic-assisted surgery has been shown to achieve more accurate cup positioning than conventional techniques [76]. To fully utilize this advantage, robotic technology should be used in conjunction with dynamic spinopelvic assessment. This integrated approach may lead to improved clinical outcomes.

## 6. Summary

In this review, we provide an overview of the current evidence on pelvic tilt changes following THA (Table 3). Pelvic orientation demonstrates a tendency toward posterior tilt. Classification- and alignment-based approaches can predict postoperative pelvic tilt with reasonable accuracy when the dominant mechanism is identified. Because prediction uncertainty remains clinically relevant, future work should prioritize risk stratification, whole-spine and bilateral hip assessment, and adaptive surgical planning. Robotic-assisted surgery in conjunction with dynamic spinopelvic assessment may lead to improved clinical outcomes.

In addition, given the potential impact of vertebral fractures on progressive sagittal alignment changes, particularly in female patients, postoperative follow-up should include evaluation and management of osteoporosis to reduce the risk of fragility fractures and secondary spinal deformity.

## Figures and Tables

**Table 1:** Summary of Included Studies According to Imaging Modality.

Study	Imaging Modality
Palit (2025) [34]	Anybody modeling system and 3D-CT fusion
Fischer (2020) [46]	CT and EOS
Fischer (2022) [58]	CT and EOS
Dennis (2023) [44]	CT and radiograph
Heckmann (2024) [59]	CT and radiograph
Konishi (2025) [29]	CT and radiograph
Lazennec (2004) [9]	CT and radiograph
Lazennec (2007) [10]	CT and radiograph
Shafiei (2025) [60]	CT and radiograph
Du (2025) [47]	CT-based matching
Fujii (2023) [61]	CT-based matching
Hamada (2023) [22]	CT-based matching
Kleeman-Forsthuber (2022) [42]	CT-based matching
Murphy (2013) [39]	CT-based matching
Nishihara (2003) [38]	CT-based matching
Suzuki (2016) [16]	CT-based matching
Tamura (2017) [21]	CT-based matching
Kamihata (2023) [40]	CT measurements and CT-based matching
Barbier (2017) [45]	EOS
Berliner (2018) [54]	EOS
Buckland (2025) [35]	EOS
Lazennec (2011) [11]	EOS
Haffer (2022) [28]	EOS
Haffer (2022) [50]	EOS
Haffer (2023) [48]	EOS
Innmann (2021) [62]	EOS
Innmann (2022) [18]	EOS
Jain (2023) [36]	EOS
Muellner (2022) [55]	EOS
Pour (2024) [23]	EOS
Sculco (2021) [30]	EOS
Tang (2024) [33]	EOS
Watanabe (2021) [51]	EOS
Windsor (2022) [31]	EOS
Pluchon (2024) [41]	Ultrasound device

Studies based exclusively on plain radiographic assessment were not included in this table.

**Table 2:** Factors Associated with Long-Term Changes in Pelvic Posterior Tilt after THA.

Study	Follow-up	Significant Factors
Chen (2023) [68]	19.1 years	Female Over 60 years of age
García-Rey (2024) [65]	≥ 10 years	Female Over 65 years of age
Hamada (2023) [22]	20 years	Preoperative large posterior pelvic tilt from supine to standing Lumbar vertebral fractures
Katsura (2022) [64]	11.8 years	Over 75 years of age * Fragility fractures *
Kromka (2025) [67]	13 years	45 to 50 years of age
Okanoue (2017) [66]	11 years	Preoperative posterior pelvic tilt Vertebral fractures
Tamura (2017) [21]	10 years	Type P **

* Tendency. ** Type P: pelvis tilted posteriorly >10° from supine to standing.

**Table 3:** Key Publications Related to Temporal Change and Prediction of Spinopelvic Alignment.

Study	Key Findings
Buckland (2025) [35]	A preoperative classification system for sagittal spinopelvic deformity was proposed.
Fischer (2022) [58]	Preoperative standing alignment enhances prediction of postoperative pelvic orientation.
Fujii (2023) [61]	Application of machine learning analysis to predict the pelvic tilt.
Hamada (2023) [22]	Pelvic tilt changes over 20 years after THA were reported.
Innmann (2022) [18]	Spinopelvic hypermobility was markedly reduced postoperatively.
Ishida (2011) [15]	Most changes in pelvic tilt occurred within the first 3 months.
Muellner (2022) [55]	Pre-existing contralateral THA influences spinopelvic mobility.
Suzuki (2016) [16]	Exhibited progressive and excessive posterior pelvic tilt over 5 years.

## Data Availability

No new data were generated or analyzed in support of this review. All data are derived from previously published studies, which are cited in the reference list.

## Supplementary Materials

The following supporting information can be downloaded at: https://www.mdpi.com/article/10.3390/jcm15062228/s1, the detailed search strategy.
