# Supplementary material for: A Narrative Review of Spinopelvic Alignment Changes After Total Hip Arthroplasty
Source: J Clin Med. 2026 Mar 15;15(6):2228. doi: 10.3390/jcm15062228 (PMC13026519; doi:10.3390/jcm15062228)
Supplement: Supplementary file 1 [file jcm-15-02228-s001.zip › JCM review Ike Table 1.pdf]

Table 1 Summary of Included Studies According to Imaging Modality

Study	Imaging Modality
Palit (2025) [34]	Anybody modeling system and 3D-CT fusion
Fischer (2020) [46]	CT and EOS
Fischer (2022) [69]	CT and EOS
Dennis (2023) [44]	CT and radiograph
Heckmann (2024) [70]	CT and radiograph
Konishi (2025) [29]	CT and radiograph
Lazennec (2004) [9]	CT and radiograph
Lazennec (2007) [10]	CT and radiograph
Shafiei (2025) [71]	CT and radiograph
Du (2025) [47]	CT-based matching
Fujii (2023) [68]	CT-based matching
Hamada (2023) [22]	CT-based matching
Kleeman-Forsthuber (2022) [42]	CT-based matching
Murphy (2013) [39]	CT-based matching
Nishihara (2003) [38]	CT-based matching
Suzuki (2016) [16]	CT-based matching
Tamura (2017) [21]	CT-based matching
Kamihata (2023) [40]	CT measurements and CT-based matching
Barbier (2017) [45]	EOS
Berliner (2018) [54]	EOS

Buckland (2025) [35]	EOS
Lazennec (2011) [11]	EOS
Haffer (2022) [28]	EOS
Haffer (2022) [50]	EOS
Haffer (2023) [48]	EOS
Innmann (2021) [72]	EOS
Innmann (2022) [18]	EOS
Jain (2023) [36]	EOS
Muellner (2022) [55]	EOS
Pour (2024) [23]	EOS
Sculco (2021) [30]	EOS
Tang (2024) [33]	EOS
Watanabe (2021) [51]	EOS
Windsor (2022) [31]	EOS
Pluchon (2024) [41]	Ultrasound device
