# Supplementary material for: A Narrative Review of Spinopelvic Alignment Changes After Total Hip Arthroplasty
Source: J Clin Med. 2026 Mar 15;15(6):2228. doi: 10.3390/jcm15062228 (PMC13026519; doi:10.3390/jcm15062228)
Supplement: Supplementary file 1 [file jcm-15-02228-s001.zip › JCM review Ike Table 2.pdf]

Table 2 Factors Associated with Long-Term Changes in Pelvic Tilt after THA

Study	Follow-up	Significant factors
Chen (2023) [63]	19.1 years	Female<br>Over 60 years of age
García-Rey (2024) [60]	≥ 10 years	Female<br>Over 65 years of age
Hamada (2023) [22]	20 years	Preoperative large posterior pelvic tilt from supine to standing<br>Lumbar vertebral fractures
Katsura (2022) [59]	11.8 years	Over 75 years of age *<br>Fragility fractures *
Kromka (2025) [62]	13 years	45 to 50 years of age
Okanoue (2017) [61]	11 years	Preoperative posterior pelvic tilt<br>Vertebral fractures

Tamura (2017) [21]	10 years	Type P **

* Tendency

** Type P: pelvis tilted posteriorly >10° from supine to standing.
