# Supplementary material for: A Narrative Review of Spinopelvic Alignment Changes After Total Hip Arthroplasty
Source: J Clin Med. 2026 Mar 15;15(6):2228. doi: 10.3390/jcm15062228 (PMC13026519; doi:10.3390/jcm15062228)
Supplement: Supplementary file 1 [file jcm-15-02228-s001.zip › JCM review Ike Table 3.pdf]

Table 3 Key Publications Related to Temporal Change and Prediction of Spinopelvic Alignment

Study	Key Findings
Buckland (2025) [35]	A preoperative classification system for sagittal spinopelvic deformity was proposed.
Fischer (2022) [69]	Preoperative standing alignment enhances prediction of postoperative pelvic orientation.
Fujii (2023) [68]	Application of machine learning analysis to predict the pelvic tilt.
Hamada (2023) [22]	Pelvic tilt changes over 20 years after THA were reported.
Innmann (2022) [18]	Spinopelvic hypermobility was markedly reduced postoperatively.
Ishida (2011) [15]	Most changes in pelvic tilt occurred within the first 3 months.

Muellner (2022) [55]	Pre-existing contralateral THA influences spinopelvic mobility.
Suzuki (2016) [16]	Exhibited progressive and excessive posterior pelvic tilt over 5 years.
