# Supplementary material for: A Narrative Review of Spinopelvic Alignment Changes After Total Hip Arthroplasty
Source: J Clin Med. 2026 Mar 15;15(6):2228. doi: 10.3390/jcm15062228 (PMC13026519; doi:10.3390/jcm15062228)
Supplement: Supplementary file 1 [file jcm-15-02228-s001.zip › JCM supplementary file.pdf]

A literature search was conducted in PubMed and Web of Science for articles published between January 2000 and November 2025 using the following search strategy: (“total hip arthroplasty” AND “change”) combined with at least one of the following terms: “spinopelvic alignment,” “pelvic tilt,” “sacral slope,” “lumbar lordosis,” or “hip–spine relationship.” Only studies published in English were considered for selection.

The PubMed search string was as follows: "total hip arthroplasty"[Title/Abstract] AND ("change"[All Fields] OR "changed"[All Fields] OR "changes"[All Fields] OR "changing"[All Fields] OR "changings"[All Fields]) AND ("spinopelvic alignment"[Title/Abstract] OR "pelvic tilt"[Title/Abstract] OR "sacral slope"[Title/Abstract] OR "lumbar lordosis"[Title/Abstract] OR "hip-spine relationship"[Title/Abstract]) AND 2000/01/01:2025/11/30[Date - Publication] AND "English"[Language]

A Topic search (TS) was performed in the Web of Science Core Collection using the following search string: TS=("total hip arthroplasty" AND (change) AND ("spinopelvic alignment" OR "pelvic tilt" OR "sacral slope" OR "lumbar lordosis" OR "hip-spine relationship")) AND Publication date is 2000-01-01 to 2025-11-30. Results were limited to English-language publications using database filters. Using these search strategy, 240 publications were retrieved from PubMed and 250 publications were retrieved from Web of Science Core Collection. After removal of duplicate records, 277 unique articles remained for further screening.

Studies were eligible if they (1) investigated changes in spinopelvic parameters following total hip arthroplasty, (2) included quantitative radiographic and/or clinical measurements. Following title and abstract screening, 219 articles met the inclusion criteria and were selected for full-text review, of which 54 met the inclusion criteria and were included in the analysis. In addition, 22 relevant studies were identified through reference list screening and supplementary searches, resulting in a total of 76 articles included in the final analysis (Fig 2).
